# Seasonal and Diel Vocalization Patterns of Antarctic Blue Whale (*Balaenoptera musculus intermedia*) in the Southern Indian Ocean: A Multi-Year and Multi-Site Study

**DOI:** 10.1371/journal.pone.0163587

**Published:** 2016-11-09

**Authors:** Emmanuelle C. Leroy, Flore Samaran, Julien Bonnel, Jean-Yves Royer

**Affiliations:** 1 University of Brest and CNRS, Laboratoire Geosciences Brest, IUEM, 29280 Plouzané, France; 2 UMR CNRS 6285 Lab-STICC, ENSTA Bretagne, 29806 Brest, France; Institute of Deep-sea Science and Engineering, Chinese Academy of Sciences, CHINA

## Abstract

Passive acoustic monitoring is an efficient way to provide insights on the ecology of large whales. This approach allows for long-term and species-specific monitoring over large areas. In this study, we examined six years (2010 to 2015) of continuous acoustic recordings at up to seven different locations in the Central and Southern Indian Basin to assess the peak periods of presence, seasonality and migration movements of Antarctic blue whales (*Balaenoptera musculus intermedia*). An automated method is used to detect the Antarctic blue whale stereotyped call, known as Z-call. Detection results are analyzed in terms of distribution, seasonal presence and diel pattern of emission at each site. Z-calls are detected year-round at each site, except for one located in the equatorial Indian Ocean, and display highly seasonal distribution. This seasonality is stable across years for every site, but varies between sites. Z-calls are mainly detected during autumn and spring at the subantarctic locations, suggesting that these sites are on the Antarctic blue whale migration routes, and mostly during winter at the subtropical sites. In addition to these seasonal trends, there is a significant diel pattern in Z-call emission, with more Z-calls in daytime than in nighttime. This diel pattern may be related to the blue whale feeding ecology.

## Introduction

**Fig 1:**
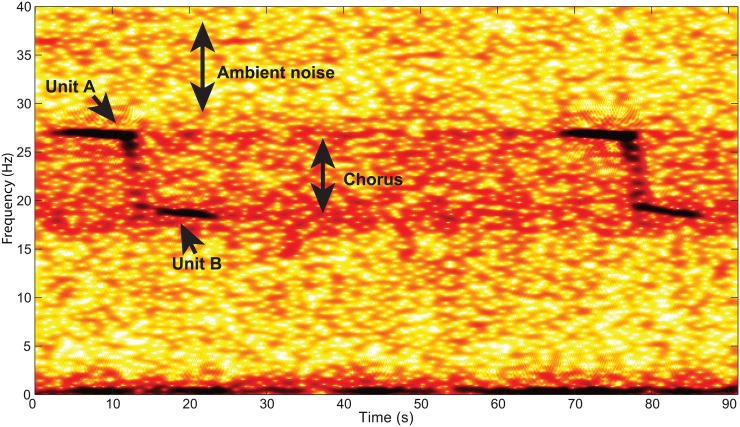
Spectrogram of two consecutive Z-calls. The noisy frequency band between 18 and 28 Hz is formed by the Antarctic blue whale and fin whale chorus.

As the preferred target of commercial whalers, the Antarctic blue whales (*Balaenoptera musculus intermedia*) were largely decimated during the 20th century. With a remaining population estimated in the mid-1970s at 0.15% of its initial size [1], Antarctic blue whales are listed as Critically Endangered by the International Union for Conservation of Nature (IUCN) [2]. Information about the population recovery and its current distribution is limited, since our knowledge about this species comes mainly from whaling data [3], and from extensive visual sighting surveys from the IDCR/SOWER program [4]. This species is found all around the Antarctic continent during austral summer [5–7], feeding on the dense patches of Antarctic krill (*Euphausia superba*), and migrates, at least for a major part of the population, to northern locations during winter. Wintering areas are believed to be off the southern African coast [5], in the eastern tropical Pacific, the Central Indian Basin [6], southwest of Australia [8, 9], and off northern New Zealand [10]. Two recent studies describe their presence in the Southern Indian Ocean [11, 12]. Acoustic data acquired near Crozet Islands in 2004 unveiled the importance of this highly productive area for two southern blue whale subspecies: the Antarctic and pygmy blue whales, with a year-round presence in the area [11]. Other acoustic records at three sites in the Southern Indian Ocean, collected in 2007, provide further evidence about the seasonal presence of blue whales in this region [12] and demonstrated that blue whale subspecies use a much wider habitat than previously proposed [5]. Because of the large and remote distribution area of the species, and of often-poor weather conditions in the Southern Ocean, passive acoustic monitoring (PAM) is probably the most efficient way to study the Antarctic blue whale, compared to traditional visual observations, that are costly, difficult, and thus sparse at high latitudes [11, 13]. For instance, during 32 years of multi-vessel visual sighting surveys around Antarctica, only 216 Antarctic blue whale encounters were reported (IDCR/SOWER program, 4112 vessel-days and 216,000 nautical miles of transect lines; [14]). On the other hand, PAM is appropriate for monitoring this species since its repertoire is composed of intense, repetitive low-frequency vocalizations, known as Z-calls from their Z-shape in the time-frequency domain (Fig 1). Z-calls are constituted of three parts: a tonal unit A, lasting about 7 to 12 s at a frequency near 28 Hz [6, 15, 16], a short downsweep of 1 to 2 s, and a tonal unit B, lasting between 7 and 12 s, at a frequency around 18 Hz. Frequency of unit A appears to be decreasing in the past decades [17–20]. Z-calls are repeated in sequences, every 40 to 70 s during several minutes to hours [6, 10, 15, 21, 22]. The highly stereotyped characteristics of Z-calls make the Antarctic blue whale presence easy to detect and monitor. In this study, Z-calls are used as a clue for Antarctic blue whale presence. However, since this call is likely to be emitted only by males, as noted for other baleen whale and blue whale (sub)species [23, 24], this acoustic indicator would be mainly representative of the presence patterns of the vocally active males. Nevertheless it appears that blue whales emit calls year-round, during reproductive as well as non-reproductive periods [7, 21, 24, 25], allowing for a year-round acoustic monitoring of this species. Unlike previous studies, generally limited in time or in geographic coverage and providing only clues about the long-term presence and distribution of Antarctic blue whales, this study uses a hydrophone network covering a wide range of latitudes and longitudes, spanning the central and south Indian Ocean (4 to 46°S, 53 to 81°E), and deployed for six continuous years from 2010 to 2015. The network consists of five to seven instrumented sites, 700 to 1500 km apart. Three sites are at the same locations as in a previous experiment in 2007 [12], which expands the period of observation.

Here, we present the results from this first six-year-long continuous acoustic monitoring of Antarctic blue whale on a broad scale in the Southern Indian Ocean. First, Antarctic blue whale Z-calls are automatically detected at each station. Second, the seasonal distribution of Z-calls and its variations across years are explored. Finally, the diel pattern of Z-call emission is examined. Results and their ecological implications are discussed in the last section.

## Materials and Methods

### Data Acquisition

**Fig 2:**
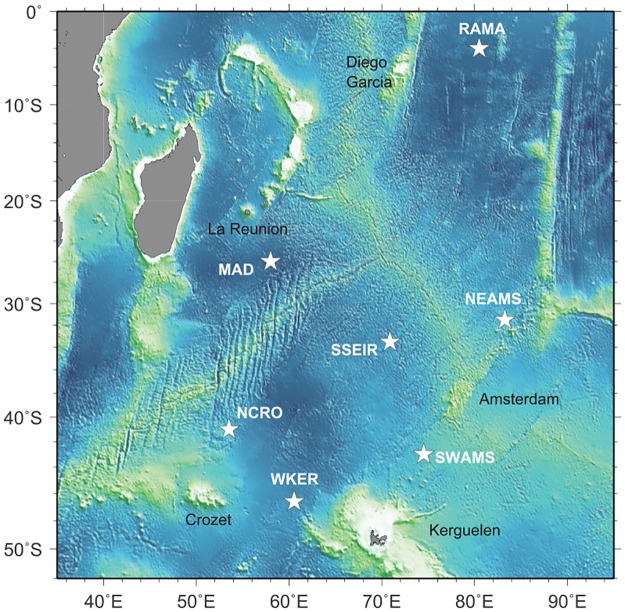
Hydrophone locations of the OHASISBIO network in the Indian Ocean (stars).

**Fig 3:**
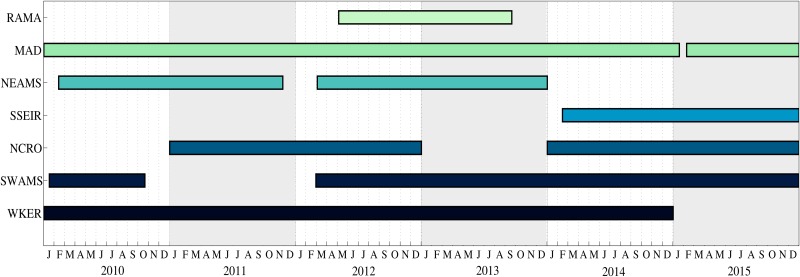
Periods of continuous recordings analysed for each site.

The hydrophone network—known as OHASISBIO—was initially deployed in December 2009 at five sites in the Southern Indian Ocean. This experiment was designed to monitor low-frequency sounds, produced by seismic and volcanic events [26, 27], and by large baleen whales. Instruments are distributed south of La Reunion Island in the Madagascar Basin (MAD), northeast of the St Paul and Amsterdam plateau (NEAMS), mid-way between the Kerguelen and Amsterdam islands (SWAMS), north of Crozet Island (NCRO) and west of Kerguelen Island (WKER). The geometry of the OHASISBIO-network slightly changed through the years, but these five sites remained the same during the whole experiment. Additional sites were temporarily instrumented, such as the RAMA site, near the Equator in the Central Indian Basin, deployed for 16 months in 2012-2013. In 2014, a new site was instrumented, just south of the Southeast Indian Ridge (SSEIR)(Fig 2). Most of the sites are equipped with a single hydrophone. However, some years, triads of hydrophones forming a triangle were deployed at some sites: in 2010 and 2011, triads with a 30 km side were deployed at NCRO and WKER sites; in 2012 and 2013, only the WKER-triad was redeployed, and in 2014 and 2015, the triad was moved to the SWAMS site, and the distance between hydrophones reduced to 10 km. Each mooring consists of an anchor, an acoustic release, and an autonomous hydrophone set to record acoustic waves continuously at a rate of 240 Hz using a 24-bit analog-to-digital conversion. Hydrophones are deployed in the axis of the sound fixing and ranging (SOFAR) channel, from 500 to 1300 m below sea surface depending on the site. The hydrophones (and data) were recovered and redeployed every year in January-February, during the annual voyages of the R/V Marion Dufresne to the French Southern and Antarctic Territories in the Southern Indian Ocean. However, in 2011, the instruments located at NEAMS and SWAMS sites could not be recovered, and remained on site until the next voyage, in 2012. The NEAMS hydrophone had enough battery to record until November 2011 (20 months), whilst the SWAMS one stopped in November 2010, after only 8 months of operation. In 2011 at WKER site, one of the three instruments was lost, and another stopped after 2 months. In 2015, the NEAMS hydrophone was lost during recovery, and in 2016, poor weather conditions prevented the recovery of WKER and NEAMS instruments. Locations of the hydrophones and the available data are listed in Table 1. Periods of continuous recordings analysed in this study are presented in Fig 3.

**Table 1:** OHASISBIO autonomous hydrophone network. The character “-” indicates continuous recordings without data recovering, “x” indicates that there is no available data. A site name followed by a number (1, 2 or 3) indicates the instruments of a triad.

Site	Geographic coordinates	2010		2011		2012		2013		2014		2015	
		Start	Stop	Start	Stop	Start	Stop	Start	Stop	Start	Stop	Start	Stop
RAMA	03 50’S, 080 30’E	x	x	x	x	05/05/12	-	-	09/19/13	x	x	x	x
MAD	26 05’S, 058 08’E	12/20/09	02/19/11	02/19/11	03/09/12	03/10/12	03/09/13	03/09/13	02/16/14	16/02/14	01/18/15	02/08/15	01/28/16
NEAMS	31 35’S, 083 14’E	02/13/10	-	-	11/25/11	03/04/12	03/04/13	03/04/13	02/10/14	x	x	not yet recovered	
SWAMS	42 59’S, 074 35’E	01/17/10	11/21/10	x	x	02/29/12	02/27/13	02/28/13	02/07/14	x	x	x	x
SWAMS 1	42 02’S, 074 36’E	x	x	x	x	x	x	x	x	02/07/14	12/02/14	01/27/15	01/20/16
SWAMS 2	42 58’S, 074 31’E	x	x	x	x	x	x	x	x	02/07/14	01/27/15	01/27/15	01/20/16
SWAMS 3	42 57’S, 074 39’E	x	x	x	x	x	x	x	x	02/08/14	01/27/15	01/27/15	01/21/16
NCRO 1	41 00’S, 052 49’E	12/25/09	01/19/11	01/20/11	01/30/12	x	x	x	x	x	x	x	x
NCRO 2	41 00’S, 053 10’E	12/25/09	01/20/11	01/21/11	01/31/12	x	x	x	x	x	x	x	x
NCRO 3	41 14’S, 052 59’E	12/25/09	01/20/11	01/20/11	01/31/12	01/29/12	02/10/13	02/12/13	01/10/14	01/11/14	01/11/15	01/11/15	01/08/16
WKER 1	46 38’S, 060 07’E	12/28/09	01/24/11	01/25/11	02/03/12	02/04/12	02/14/13	02/15/13	01/15/14	x	x	x	x
WKER 2	46 34’S, 060 31’E	12/28/09	01/25/11	x	x	02/05/12	02/15/13	02/17/13	10/23/13	01/15/14	01/01/15	not yet recovered	
WKER 3	46 50’S, 060 24’E	12/28/09	01/25/11	01/25/11	03/10/12	02/03/12	02/16/13	02/16/13	01/16/14	x	x	x	x
SSEIR	33 30’S, 070 52’E	x	x	x	x	x	x	x	x	02/13/14	02/04/15	02/05/15	01/18/16

### Acoustic data processing

Except for the NCRO data in 2010 and 2013, all the data are exploitable. The analysis of the NCRO-triad in 2010 and in 2013 is hindered by a high noise-level probably generated by the mooring line and occurring in the same frequency band than whale calls.

#### Call detection

For such a large amount of acoustic data, we resorted to an automatic Z-detector based on a subspace-detection algorithm [28]. The main advantage of this detector is that it does not suffer from the inherent limitations of the classical correlation-based detectors. In particular, it does neither require an *a priori* fixed template nor a user-chosen detection threshold. Indeed, the algorithm has an adaptive detection threshold, which depends on the ambient noise level, which ensures a maximum false-alarm probability of 3%, even in presence of interfering signals. The algorithm models the Z-call shape with a logistical function (i.e. a mathematical equation which, when plotted, has a Z-shape), which requires four parameters: *U* and *L* to set the upper and lower frequencies of the model (i.e. frequencies of units A and B), a growth rate *α*, set to 2.1, and *M*, the time shift of the logistic function (related to unit A duration), fixed to 10.23. The frequency parameters *U* and *L* are adapted depending on the year of the treated recordings. Indeed, the frequency of the unit A of Z-calls appears to be decreasing in the past decades [17–20], at an estimated rate of 0.135 Hz per year [19]. The Z-detector is robust to frequency variations between calls and to intra-annual changes, but to ensure this flexibility while limiting the number of false detections, the frequency interval into which the model can vary is limited to 0.5 Hz. Three values define the frequency parameters *U* and *L*. Because the unit B frequency remains stable over the years, the same parameters *L* are used for any year of data: L1 = 19 Hz, L2 = 18.75 Hz and L3 = 18.5 Hz. Parameters for Unit A are in Table 2.

**Table 2:** Parameter *U*, defining the unit A frequency to model the Z-call for each year of data.

	U1	U2	U3
**2010**	26.75 Hz	26.5 Hz	26.25 Hz
**2011**	26.75 Hz	26.5 Hz	26.25 Hz
**2012**	26.60 Hz	26.35 Hz	26.10 Hz
**2013**	26.50 Hz	26.25 Hz	26.00 Hz
**2014**	26.30 Hz	26.05 Hz	25.80 Hz
**2014**	26.05 Hz	25.80 Hz	25.55 Hz

#### False detection discrimination

In any acoustic database, interferences of various types can occur (e.g. airguns, other baleen whale calls, seismic events, etc). Yet, the number of false detections generated by such interferences are limited due to the Z-detector characteristics [28]. Nevertheless we develop a method for removing potential false detections. For each detection, the Z-detector output the frequency of the signal at its maximum amplitude. If this frequency departs from the frequency of unit A of the Z-call, which is the most energetic part of the call, it is likely that the detection is not a Z-call, but rather a false detection. Thus, we exclude all the detections with a frequency above and below the selected frequency for unit A for the processed year.

#### Ambient noise measurement

To measure the evolution of the ambient noise in our study area over the years, and its possible impact on the number of detected calls, the ambient noise level is calculated in the 40–60 Hz frequency band for each station and each year. This frequency range is dominated by distant shipping, seismic airgun signals, and biological sounds [29]. This band does not contain Antarctic blue whale calls, or very short ones (such as D-calls). Ambient noise level is estimated over 300s-windows with 0.0018 Hz-bins, averaged per month, and reported in decibels (dB re 1 μPa^2^/Hz).

#### Chorus to Noise-without-chorus Ratio (CNR)

In the presence of numerous Antarctic blue whales, the overlay of distant calls creates a “chorus” (Fig 1) that sometimes makes impossible the identification of individual calls. This chorus could indicate that whales are in the area, but not close enough to the hydrophone to be detected. The power of this chorus and more precisely, the Chorus to Noise-without-chorus Ratio (CNR) may thus usefully complement the detection results, since a lack of detection does not necessarily mean an absence of calling whales. To estimate this CNR, the chorus level is calculated in a frequency band set to 25.5–26.8 Hz for 2010 and 2011 datasets; 25.5–26.7 Hz for 2012 data; 25.5–26.5 Hz for 2013 and 2014; and 25.5–26.1 Hz for 2015. These bands are chosen to take only into account the Unit A of Z-calls and to avoid the 20-Hz fin whale pulses very abundant in our recordings. The 20-Hz fin whale pulses are centered around 20 Hz, but begin at around 15 Hz and end at around 30 Hz, with a maximum amplitude at about 18 Hz. This chorus level (in dB re 1 μPa^2^/Hz) is then subtracted from the noise level in the 30–33 Hz frequency band, and averaged per month. Note that the frequency band of noise used to estimate the CNR is different from the frequency band used for the noise level estimation (40–60 Hz). Indeed, this range is chosen to be as close as possible to the chorus, and not too wide compared to the chorus frequency range.

### Detection results analysis

#### Statistical analysis of detection results

As described previously, depending on the year, some sites were instrumented with hydrophone triads. The monthly distributions of detection results obtained for each hydrophone of a triad are compared, in order to check if they differ between instruments 30 km or 10 km apart. This comparison will tell 1) whether any instrument is representative of the triad, i.e. whether the analysis of only one instrument of a triad does not introduce any bias, and 2) the relevance of detection numbers for characterizing the Antarctic blue whale presence at a large spatial scale.

To enable the comparison between years and stations since some years of recording are incomplete, the number of detections per day is estimated using a Generalized Linear Mixed Model (GLMM). This GLMM is performed using a negative binomial distribution, which is suitable for overdispersed count data, using month and year taken as random effects [30].

To test whether seasonality varies from year to year at a given station, monthly distributions of detections are normalized by the total number of detected Z-calls in the given year. The normalization makes the observation independent from variations in the absolute detection numbers between years and emphasizes their seasonality.

Finally, to study the diel calling pattern of Antarctic blue whales, Z-call detections are sorted into four light regimes based on the altitude of the sun: dawn, light, dusk and night. Dawn hours start when the sun is 12° below the horizon (i.e. morning nautical twilight) and end at sunrise; light hours are between sunrise and sunset; dusk is between sunset and the evening nautical twilight; and night hours are between dusk and dawn, when the altitude of the sun is less than -12°. Daily hours of sunset, sunrise and nautical twilights were obtained from the United States Naval Observatory Astronomical Applications Department Web site (http://aa.usno.navy.mil) for each year and each site location. The daily number of Z-calls in each light regime is calculated, and divided by the duration of the corresponding light period for a given day, to account for the difference of duration between the four light regimes and their seasonality. The resulting normalized detection rates (in detections/hr), for each light regime and each day, are then adjusted by subtracting the mean number of detection per hour of the corresponding day [31, 32]. These adjusted means of Z-calls per light period are then averaged over the seasons of Z-call main presence, depending on the site location. Seasons are defined by the dates of the solstices and equinoxes for each year.

Distribution of Z-calls per site, per year or month or light regime are not normally distributed. So to compare distributions between sites of a triad, or between years or light regimes at a same site, we use Friedman or Kruskal-Wallis tests [33]. In cases of significant differences between distributions, additional Wilcoxon pairwise comparison tests with Bonferroni correction are used [34, 35].

Statistical analyses were performed using R [36], and GLMM was run using STAN called from R with the package RStanArm (http://mc-stan.org/) [37].

## Results

### Ambient noise level

**Fig 4:**
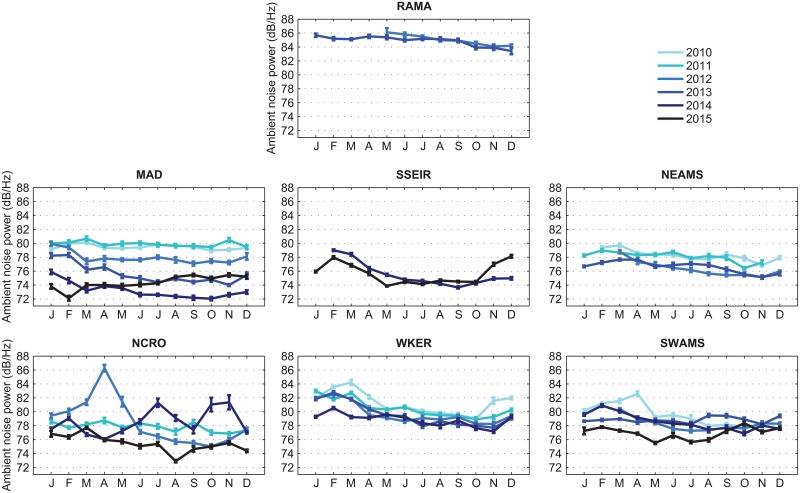
Ambient noise level in the 40–60 Hz frequency band for each available year at each site.

Since a high ambient noise level would decrease the signal-to-noise ratio (SNR) of calls, and thus the detection probability (e.g. [38]), we examine the ambient noise level in the 40–60 Hz frequency band for each available year of data at each station (Fig 4). The ambient noise level is higher at RAMA (around 85 dB/Hz) than at the other sites, which all display a decreasing noise level between 2010 and 2015, especially at MAD and NEAMS. Aside some peaks (e.g. in April 2012 and October-November 2014 at site NCRO, or April 2010 at SWAMS), the levels of noise are fairly constant throughout the year at each site, which ensures that variations in Z-call detection are not artifacts of the ambient noise level. A further analysis of the ambient noise level can be found in [39].

### Inside triad comparison

Kruskal-Wallis comparison tests reveal no significant difference between monthly detections at each instrument of a triad, either for the 30 km-triad, or for the 10 km-triad. Thus, we assume that one instrument per triad is representative of the site. We selected the hydrophone according to the quality, the continuity and length of the recordings. For the WKER-triad, hydrophone 1 (WKER 1) was chosen for 2010 to 2013. For the NCRO-triad, hydrophone 2 (NCRO 2) was chosen in 2011, and hydrophone 3 (NCRO 3) in 2012. Finally, for 2014 and 2015, recordings of the hydrophone 2 of the SWAMS-triad were chosen.

In addition, this comparison confirms the relevance of assessing the presence of Antarctic blue whales using detected calls from sparse and distributed hydrophones. Indeed, significant differences in Z-call detections between instruments only 30 km or 10 km apart would have meant that the Z-call detection range is greatly lower than expected [40, 41], making the detection of calls only relevant locally.

### Site frequentation and inter-annual variation

Automated detection results show that Antarctic blue whale Z-calls are detected at every OHASISBIO sites and for each available year of data, except at RAMA, where no Z-call is detected in the 16 months of recording. A total of 252,333 Z-calls are detected at MAD station across the 6 years of recordings (2010–2015), 161,885 Z-calls at NEAMS station throughout 4 years of data (2010-2013), 191,939 Z-calls at SWAMS site in 5 years (2010, 2012-2015), 111,576 Z-calls at NCRO for the 4 years of exploitable recordings (2011-2012, 2014-2015), 297,451 Z-calls at WKER during 5 years (2010-2014), and 59,506 Z-calls at SSEIR for the two years 2014-2015.

**Fig 5:**
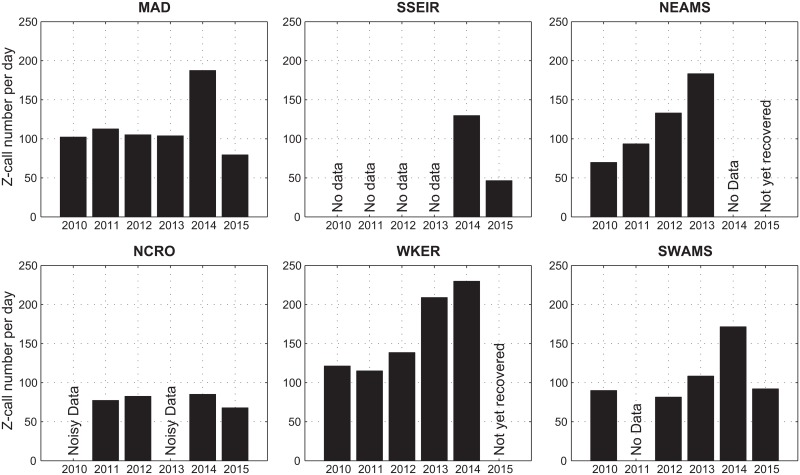
Number of Z-calls per day for each available year at each station.

Fig 5 presents an estimate of the number of detected Z-calls per days of recordings for each year of data at each station. This metric is necessary since some years of data are not complete. Globally, NCRO station shows a lower number of detections (below 85 Z-calls/day) than the others, as SSEIR in 2015 (around 47 Z-calls/day). Moreover, 2014 seems to be an abnormal year, with a higher number of detections than the other years, which is especially obvious at MAD station. It could be argued that this higher detection rate is due to a lower ambient noise level in 2014. Still, it can be noticed that from 2010 to 2013, the noise level at MAD decreased by around 2 dB every year whilst the number of detection remained constant. In addition, SWAMS shows a constant noise level throughout the years, but a sharp increase in the number of calls in 2014. So we conclude that the 2014 increase in the detection rate is significant and not solely imputable to a decrease in the ambient noise level.

Finally, results show no homogeneous pattern. Indeed, the detection number varies between years and stations, and no overall trend can be observed on all sites, neither global increase nor decrease of the total detection number along the years.

### Seasonal patterns

**Fig 6:**
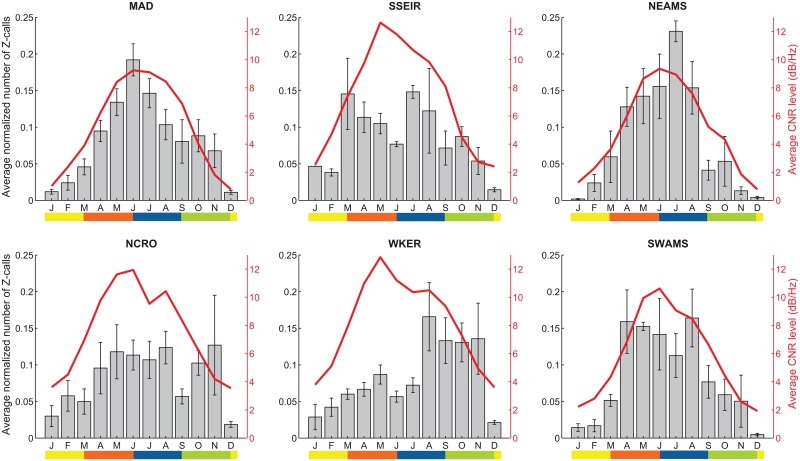
Normalized number of Z-calls detected per month averaged over the available years of data for each station, and corresponding Chorus to Noise-without-chorus Ratio (CNR) level (red curves). The color bar represents the seasons (yellow: summer; brown: autumn; blue: winter; green: spring).

For MAD, NEAMS, NCRO, WKER and SWAMS sites, statistical comparisons show no significant difference among the normalized monthly distributions of Z-calls between years (Friedman tests, respectively for MAD, NEAMS, NCRO, WKER and SWAMS, *Friedman* *chi* − *squared* = 0.8; 1.13; 1.84; 3.1; 0.26; all with a probability *p* in favor of the null hypothesis > 0.05). For SSEIR site, a Wilcoxon test for paired data (*V*) shows no significant difference between the two years of data (Wilcoxon paired test, *V* = 34, *p* = 0.96). This allows averaging the normalized monthly distributions over the available years for these sites and to compare them with the corresponding averaged Chorus to Noise-without-chorus Ratio (CNR) levels (Fig 6).

At these six sites, Z-calls are recorded throughout the year, but with strong seasonal patterns that differ between locations. At MAD station, Z-calls are mainly detected from April to November (austral autumn to spring), with a detection peak in June (during winter). The mean CNR fits the average monthly distribution, and thus confirms the information provided by the detections. A very low number of Z-calls is detected during austral summer, consistent with the very low CNR level (around 1 dB/Hz). This is also the case for the NEAMS station. At this station, Z-calls are also detected from autumn to spring, with a more important presence from April to August (from late autumn to early spring), and a detection peak in July. Here again, the averaged CNR ratio fits pretty well with the detection number.

Only two years of recordings are currently available at the SSEIR site, deployed since 2014. Z-calls are mainly detected from March to November (autumn to spring), with a higher presence in the beginning of autumn and in winter. However there is no simple pattern, and this distribution differs from the CNR level, which reaches its maximum in May and progressively decreases until November.

Seasonality at NCRO station is also unclear. Z-calls are mostly present from April to November (autumn to late spring), with no detection peak. The CNR level does not match the detection numbers: beginning at a higher summer level than in the previously described stations (around 3 dB/Hz), it shows a level increase in autumn, until May-June, a slight decrease in June-July, a small increase from July to August, and then a steady decrease until December. Furthermore, the number of detected Z-calls during the austral summer, although lower than the rest of the year, is greater than for the northernmost stations, which is consistent with the higher CNR level observed at this period. This last observation also stands for WKER, where Z-calls are detected throughout the year, with a main presence from August to November, during spring. Despite the lower presence of Z-calls in autumn, the CNR level sharply increases from February to May, then decreases until July, levels in August, and finally decreases until December. Visual inspections of some of these periods with high CNR level and low detection numbers indicate the presence of highly degraded signals that cannot be called “Z-calls” anymore (i.e. an experimented human perator would not have annoted them as Z-calls). Thus, such a low detection number is not due to a large miss-detection number. Finally, at SWAMS station, Z-calls are detected from March to November, with a strong increase in the detection number in April (mid-autumn), and again in August (late winter), both followed by a progressive decrease of Z-calls. During the summer months, very few Z-calls are recorded. The CNR confirms these observations, with a level increase (initially at about 2 dB/Hz) from March to June, a decrease until July and August, followed by a steep decrease until December.

### Diel pattern

**Fig 7:**
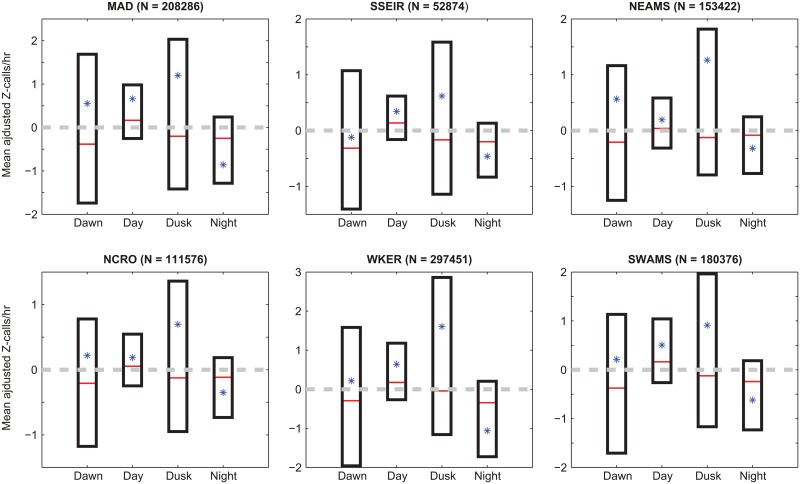
Boxplot of mean-adjusted number of detections per hour during four light regimes, averaged over available years of data for each station and over seasons of Antarctic blue whale presence of the corresponding station (autumn, winter and spring for MAD, NEAMS, SSEIR and SWAMS; the entire year for NCRO and WKER). Lower and upper bounds of boxes represent lower and upper quartiles, respectively. Red lines are median values and asterisks are mean values. Note that means (asterisks) sometimes differ from median due to many outliers, not shown in the graphic for more readability. *N* is the total number of detections during the seasons of presence.

Detection rates per light regime were averaged over the seasons of Antarctic blue whale presence, depending on the site. At MAD, SSEIR, NEAMS and SWAMS, they were averaged over autumn, winter and spring; and over the entire year at NCRO and WKER (see Seasonal patterns). For each station, the null hypothesis that the call rate is the same for the four light regimes is rejected by Kruskal-Wallis tests (*KW*) (respectively for MAD, SSEIR, NEAMS, NCRO, WKER and SWAMS: *KW* = 195.1; 98.9; 43.6; 101.2; 342.4; and 184.9; all with a probability *p* < 0.001). Wilcoxon pairwise comparison tests (*W*) show that for all stations, day and night periods are significantly different from one another, with more Z-calls emitted in daytime than in nighttime (respectively for MAD, SSEIR, NEAMS, NCRO, WKER and SWAMS: *W* = 1,270,400; 208,642; 632,154; 997,499; 2,131,618; 1,127,672; all with *p* < 0.001) (Fig 7). For dawn and dusk periods, there is an important variance in the calling rate for both light regimes, with a great number of outliers, which explains the large difference between mean and median. Thus no trend can be found for these intermediate periods.

## Discussion

In 2007, Branch et al. [5] reviewed existing datasets of catches, sightings and acoustic records, and concluded that, despite records in the northern Indian Ocean, along the Australian coast, and south of 35°S, blue whales were absent in the south-central Indian Ocean. In 2010 and 2013, Samaran et al. [11, 12] showed, however, that Antarctic blue whales are in fact present in this area, especially during winter months. Furthermore, these authors found that the central and southern Indian Ocean could be a year-round habitat for at least four populations of blue whales, including the Antarctic subspecies. Although this evidence changed our view of the Antarctic blue whale seasonal distribution in the Southern and Indian oceans, they are based on limited sites and years of observation. Our extended data set, spanning six years and a wide range of latitudes and longitudes in the central and southern Indian Ocean provides a more complete view of the Antarctic blue whale presence and seasonality in this region and how they evolve through time.

### Ambient noise

A clear higher level in the ambient noise is observed at the RAMA station, in the Central Indian Basin, than in the rest of the OHASISBIO sites; it is likely due to a greater contribution of shipping noise at these latitudes [39]. Contrary to what is expected and generally observed [42], the deep water ambient noise level measured at our stations in the 40–60 Hz frequency band is decreasing from 2010 to 2015, especially at MAD and NEAMS sites. This notable decrease is not totally surprising, since a similar observation is made in the South Atlantic Ocean [43]. However, at Diego Garcia Island, ambient noise in the 40–60 Hz frequency-band has been increasing in the past decades [29]. Further analyses of these long-term inter-annual changes in the ambient noise are beyond the scope of our study. Our purpose, here, is to make sure that changes in the number of detected Z-calls are unrelated to changes in the ambient noise level. Indeed, looking at the inter-annual variation of the total number of Z-calls per day throughout the years, it can be observed, for example at MAD station, that despite the ambient noise level decreasing over the years, the detection numbers remain quite stable, except in 2014 where it is higher, but not linked to any major decrease of the ambient noise. Furthermore, the observed seasonality in the number of Z-calls is also not linked to the intra-annual variations in the noise level. As an example, at MAD and NEAMS stations, Z-calls are mainly detected during austral winter and are scarce during summertime, whilst the ambient noise level remains stable throughout the year. Thus, the observed seasonal patterns do reflect variations in the whale presence, and are not due to better or lesser performances of our Z-detector in a varying ambient noise.

### Site frequentation

Antarctic blue whale Z-calls are detected at every site of the network, except at RAMA. Their absence at 4°S is not surprising, since Antarctic blue whales would not migrate much above subtropical latitudes [6]. The year-round presence of Z-calls at all other sites, the consistency of these detections over the years and the important number of detected calls demonstrate that the south-central and the southern Indian Ocean is a wintering area for the Antarctic Blue whales, as previously suggested [11, 12]. The number of detected Z-calls per year is quite important at every site, indicating that all sites are attended, and that the entire region covered by our network is within the distribution area of Antarctic blue whales. The global attendance is however lesser at NCRO station, which is surprising, given the very high number of Z-calls reported near Crozet in 2004-2005 [22]. In this latter study, the monthly number reached a maximum of about 20,000 Z-calls and was usually comprised between 5,000 and 10,000 calls for most of the other months, whereas over all our years of data, this number reaches a maximum of about 10,000 Z-calls and is below 5,000 for most of the other months. The location near Crozet Islands of the hydrophones used in [22] may explain these differences, since the shallow environmental conditions off Crozet Islands [11] would make the habitat more favorable than in the open ocean. But it is also possible that changes in these conditions and/or in the attendance of the area occurred since 2005. SSEIR station is also globally less attended than the other sites, meaning that its location is less favorable in terms of environmental conditions, but two years of data are insufficient to draw any definitive conclusion. Additional records from the coming years will help refining this observation.

The species thus seems to spread over a wide range of longitudes in the subtropical and subantarctic waters of the Indian Ocean, since Z-calls have been recorded off Australia [6, 8, 9, 12]. Nevertheless, the number of calls reported in these studies is much lower than at our stations. Indeed, Stafford et al. [6] detected a maximum of 700 Z-calls in a single month, when it can reach up to about 19,000 detections at our stations. Tripovich et al. [9] detected 15,064 Z-calls over 15 months, that average to about 33 calls per day, whereas the lowest number of detections per day in our data set is about 47. Keeping in mind that the detection methods are different between studies, and that the number of detected calls depends on the detection range of each station, it can be carefully assumed that Antarctic blue whales are less present in the eastern part of the Indian Ocean and seem to prefer the west and central parts. Extending acoustic monitoring in the eastern longitudes would help refining this result.

The spread of vocalizing individuals in the study area changes from year to year, since the annual number of detections varies between years at a station and non-homogeneously among stations. It suggests that, given that the migration movements govern the whale attendance at different locations, these movements vary from year to year. In other words, one station can be more frequented one year, and less the following year. Thus, individuals or groups of individuals do not always use the same migration routes and/or change of wintering area between years, as noticed during commercial whaling [6]. Environmental conditions could be responsible for these changes, making sites more or less suitable. Although it was traditionally thought that baleen whales fast during migration and at breeding grounds, wintering areas seem to be determined by the availability and abundance of krill during the austral winter [5, 44]. Analyzing how the environmental conditions change over the years may help exploring this hypothesis and understanding, for instance, the large increase of detected calls in 2014. It is also possible that changes in migration routes reflect changes of breeding areas, which would lead to a genetic mixing, given that the stereotyped Z-calls are likely emitted by solitary travelling males and may have a reproductive function, by analogy with the eastern North Pacific blue whale calls [24].

Furthermore, from this non-homogeneous variation in the annual detections between stations, it is impossible to infer an evolution in the overall population size or at least of its calling part, under the basic assumption that Z-call numbers are a proxy for the number of individuals [12]. These results emphasize the importance of multi-site studies, and the danger of hasty conclusions about the evolution of a population size with a single site. For example, looking at the Z-call numbers at NEAMS site only would lead to the conclusion that the population size is growing over the years, whereas looking at MAD or NCRO stations, the conclusion would be that the population is stagnating.

Differences in the detection numbers between sites may also reflect differences in the detection range. Z-call detection range has been estimated at up to 200 km [40, 41], but is likely to vary with the environmental conditions surrounding the hydrophone, according to the latitude and season (e.g. [45–47]). Detection range will also depend on the noise level, the source level and the depth of the vocalizing whale. These parameters are poorly known and small variations in their estimate greatly impact the detection range. Simple Monte-Carlo simulations, assuming realistic input parameters, show that the detection range can vary from a few hundred kilometers to nearly 1000 km (Rémi Emmetière, personal communication 2016). Given the large uncertainties in predicted detection ranges (e.g. [45]), we believe that normalizing the detection numbers by these distances would introduce a more arbitrary bias than assuming equal (unknown) detection ranges for all sites at all seasons.

### Seasonal patterns

Despite the fact that individuals could change their migration routes and wintering areas, and spread differently in the study area from one year to another, strong seasonal patterns govern their presence at each site. Such migration patterns, occurring between low-latitude breeding grounds and high-latitude feeding grounds, have been early noticed from visual observations and whaling data (e.g. [3, 48]) and recently confirmed by passive acoustic monitoring in the Indian and Southern oceans [6, 7, 11–13]. The current study shows that despite an inter-annual variation in the total number of Z-calls per year, these seasonal patterns are stable between years. Furthermore, our results are consistent with the patterns previously observed in 2007 [12] for the MAD, NEAMS and SWAMS sites, suggesting that no significant change in the Antarctic blue whale seasonal presence occurred in 8 years.

At all stations (except RAMA), Z-calls are present year-round, but are considerably less numerous during summer months. In summer, it is believed that Antarctic blue whales are mainly in the Antarctic feeding grounds [5, 12], where numerous Z-calls are detected [7, 21, 25]. At our northernmost sites, MAD and NEAMS, the number of Z-calls increases from the mid-autumn to reach its maximum during austral winter, then progressively decreases until late spring, meaning that the vocalizing part of the Antarctic blue whale population progressively arrives at these low latitudes, on their way to or settling at wintering grounds, and leaves them in the spring to go south. The progressive increase and decrease of the monthly numbers of Z-calls may reflect the observation that migrations are more in the form of a procession than of a large school movement [3]. Following the hypothesis that our MAD and NEAMS stations are on the migration route to wintering areas, it would mean that Antarctic blue whales migrate further north. Z-call detections near Diego Garcia Island [6] show peaks in May and June for Diego Garcia North (6.3°S, 71.0°E) and in July for Diego Garcia South (7.6°S, 72.5°E), indicating that some Antarctic blue whales reach these very low latitudes. However, these detection peaks are less than 750 calls, while at MAD and NEAMS stations, over the 4 (at NEAMS) to 6 (at MAD) years of data, they range from 4,000 calls for the weakest peak, to about 18,800 calls for the highest one. Although the detection methods differ, we assume that their performances cannot be that different. Thus, it can be concluded that out of the number of Antarctic blue whales detected at MAD or NEAMS station, only few individuals migrate to lower latitudes such as Diego Garcia. Wintering at such northern latitudes would explain the passage of whales and hence the important number of Z-calls until late spring near MAD and NEAMS on their way to the Antarctic feeding grounds. Nonetheless, this observation near Diego Garcia being from 2002-2003 [6], it may also be possible that Antarctic blue whale seasonal presence has changed since then. The exact location of the Antarctic blue whale breeding areas is still not precisely known [44]. It appears therefore that, contrary to other baleen whale species such as humpback, gray or right whales, blue whales seem to spread out very widely across the oceans for breeding. Complementing earlier observations [6, 12], our data suggest that wintering, possibly breeding, grounds encompass all latitudes between 26°S (MAD) or 31°S (NEAMS) and up to a northern limit at 7°S (Diego Garcia), since no Z-calls are recorded at RAMA (4°S).

The limited dataset (2 years) at the SSEIR station suggests that this site is located on a migration path from/to wintering areas north of MAD and NEAMS latitudes and Antarctica. It would explain the larger occurrence of Z-calls in autumn, late winter and spring than in summer.

For the three subantarctic stations, the CNR patterns, which increase in autumn, decrease during winter and increase again in spring indicate that in this areas, whales are mainly present during autumn and spring, matching respectively with their northward and southward migrations, and are less present in winter, when they are at northern latitudes, in the wintering area. At SWAMS, Z-calls are mainly detected in autumn, then in early spring, suggesting the passage of blue whales near the site in autumn to wintering areas, and in spring to feeding areas. The progressive decrease of detected calls along the seasons could indicate a time-lagged migration [49]. At WKER, Z-calls are mainly detected in spring, suggesting that the site is on the southward migration route; their limited number in autumn, despite a very high CNR level, suggests that whales are not close enough to WKER to be detected, but are not totally absent of the area. The northward migration route could thus be located out of the Z-call-detection range. The CNR detection range, even more than the Z-call detection range, is not precisely known. Adding Z-calls from several individuals at various distances to form a chorus is also difficult to simulate, and its detection range is thus hard to assess. However, it is safe to assume that the chorus detection range is larger than the Z-call detection range, providing a broader acoustic “view” than individual Z-calls, and is smaller than the distance between each site. Even if not fully understood, CNR provides a useful metric for interpreting Z-call numbers and tempering any conclusion on the absence or presence of Antarctic whales from Z-call detections only (SSEIR and WKER are good examples). Finally, the NCRO station is the most peculiar. Antarctic blue whales are present almost throughout the year, with no obvious pattern in the detection number. Our results are consistent with those of Samaran et al. [49], who suspected a mid-latitude Antarctic blue whale wintering area, or a time-lagged migration.

According to the migration paradigm described earlier [3], Antarctic blue whales winter in subtropical to subantarctic latitudes and feed in the summer in the high latitudes near Antarctica. Our data confirm this general picture, however Z-calls are also recorded in the summer at all sites. Conversely, Z-calls are recorded during the winter months off Antarctica [7, 21, 25]. This observation means that parts of the population of whales remain and probably feed in the subtropical to subantarctic latitudes in the summer as well as in the high latitudes during winter. Their migration pattern thus looks more complex with time-lags between individuals, perhaps depending on their conditions (sex, age, etc). Off South African west coast, most of the blue whales caught during whaling were immature juveniles, as well as pregnant females, suggesting that this part of the population choose not to migrate and stay in the subtropical and subantarctic waters [50]. This would explain the continuous flux of vocalizing whales year-round in the study area. Furthermore, WKER and NCRO both present a higher number of Z-calls during summer months than the other sites, suggesting that the Crozet and Kerguelen plateaus are favorable feeding areas for individuals that do not migrate south [12].

### Diel pattern

Calling rates of Antarctic blue whale follow a diel pattern, with significantly less calls emitted during nighttime than during daytime. In the eastern tropical Pacific, blue whales emit more stereotyped vocalizations at night [31, 32]. These studies showed an anti-correlation between vocalizing and feeding activities, assuming that during feeding lunges, blue whales are unable to vocalize. Indeed, blue whales cannot produce their long-duration, low-frequency and high-level calls at depth greater than 40m [51, 52]. Furthermore, since feeding and singing are not mutually compatible, blue whales could use their travel time between prey patches to signal them to potential mates, with little extra energy expenditure [24]. At our latitudes, the main prey of blue whales are especially krill (*Euphausia vallentini* and *Euphausia frigida*), as well as myctophids (*Myctophum punctatum*) [49, 53]. Although the diel migration of these species is not well documented in our study area, they are known to migrate at lower depth and to be more diverse and dense at night [54–56]. This would explain the lesser number of calls of Antarctic blue whales at night and validate the trade-off between feeding and vocalizing activities formulated in previous studies [24, 31, 32]. However, off the Australian coast, the Antarctic blue whales are found to vocalize more during the night [9], but no explanation is provided. It could be because they feed on other species of prey, with different migration pattern, given that there is considerable variation between krill species behaviors [57]. In addition, feeding habits of blue whales remain uncertain; they have been observed to feed on krill when it swarms at the sea surface, and also in deep dives [58]. Furthermore, linking the observed diel calling pattern with the availability of prey implies that blue whales feed not only during summer months, but also during their migration. This hypothesis is consistent with the fact that the blue whale distribution in winter seems also influenced by feeding opportunities [5].

## Conclusion

This study, based on an analysis of Antarctic blue whale Z-calls, provides a more comprehensive picture about this whale species distribution in the Southern Indian Ocean, than in previous studies [11, 12]. Our extended acoustic dataset spanning up to 6 years, 42 degrees in latitude and 28 degrees in longitude shows 1) that Antarctic blue whales are present year-round in subantarctic and subtropical latitudes of the Indian Ocean, with a lesser presence in the austral summer, 2) that the distribution of Antarctic blue whales is highly seasonal, 3) that the seasonal patterns differ between sites but remain stable over the years, 4) that their wintering area may expand from 26°S and 7°S, and 5) the existence of a diel pattern in the emission of Z-calls, more frequent in daytime than in nighttime. Z-calls are mainly detected during autumn and spring at the subantarctic locations, suggesting that these sites are on the Antarctic blue whale migration routes, and mostly during winter at the subtropical sites, supporting the presence of a wintering and possibly breeding area at these latitudes. An analysis at a finer temporal scale is nevertheless needed to understand the inter-annual variation in sites attendance in the light of environmental condition changes, and to link the observed patterns of whale presence and call emission with environmental parameters such as sea surface temperature, chlorophyll concentration or presence of krill and myctophids in the instrumented areas. This paper also highlights the value of a multi-year and multi-sites acoustic monitoring and the caution that must be exerted when interpreting data from a single site over a limited period, for instance in terms of population evolution. Our results further demonstrate the performances of an automated Z-detector and the usefulness of jointly monitoring the Chorus to Noise-without-chorus Ratio. It would be worth complementing this study with acoustic records from the feeding areas of Antarctic blue whales, off Antarctica, and using a similar approach to be fully comparable.
